# Integrative Analysis of Methylation and Copy Number Variations of Prostate Adenocarcinoma Based on Weighted Gene Co-expression Network Analysis

**DOI:** 10.3389/fonc.2021.647253

**Published:** 2021-04-01

**Authors:** Yaxin Hou, Junyi Hu, Lijie Zhou, Lilong Liu, Ke Chen, Xiong Yang

**Affiliations:** ^1^Department of Urology, Union Hospital, Tongji Medical College, Huazhong University of Science and Technology, Wuhan, China; ^2^Shenzhen Huazhong University of Science and Technology Research Institute, Shenzhen, China

**Keywords:** bioinformatics analysis, prostate adenocarcinoma, biomarker, prognosis, therapeutic target

## Abstract

Prostate adenocarcinoma (PRAD) is the most pervasive carcinoma diagnosed in men with over 170,000 new cases every year in the United States and is the second leading cause of death from cancer in men despite its indolent clinical course. Prostate-specific antigen testing, which is the most commonly used non-invasive diagnostic method for PRAD, has improved early detection rates in the past decade, but its effectiveness for monitoring disease progression and predicting prognosis is controversial. To identify novel biomarkers for these purposes, we carried out weighted gene co-expression network analysis of the top 10,000 variant genes in PRAD from The Cancer Genome Atlas in order to identify gene modules associated with clinical outcomes. Methylation and copy number variation analysis were performed to screen aberrantly expressed genes, and the Kaplan–Meier survival and gene set enrichment analyses were conducted to evaluate the prognostic value and potential mechanisms of the identified genes. Cyclin E2 (*CCNE2*), rhophilin Rho GTPase-binding protein (*RHPN1*), enhancer of zeste homolog 2 (*EZH2*), tonsoku-like DNA repair protein (*TONSL*), epoxide hydrolase 2 (*EPHX2*), fibromodulin (*FMOD*), and solute carrier family 7 member (*SLC7A4*) were identified as potential prognostic indicators and possible therapeutic targets as well. These findings can improve diagnosis and disease monitoring to achieve better clinical outcomes in PRAD.

## Introduction

Prostate adenocarcinoma (PRAD) is one of the most common neoplasms worldwide, ranking 4th among all cancer types in both sexes with an incidence of 7.1% (1). In the United States, PRAD is the most prevalent cancer in men and is estimated to have caused more than 30,000 deaths in 2020 (1, 2).

Cancer was previously considered as a genetic disease, but there is considerable evidence that epigenetic changes contribute to tumorigenesis and tumor progression (3–5). DNA methylation is the most widely studied epigenetic modification in both non-neoplastic and neoplastic diseases including PRAD (6). The methylation of CpG islands, which are often located in the gene promoter, results in transcriptional silencing (7). Recently, methylation of enhancer regions has also been shown to play an important role in regulating gene expression (8, 9). DNA methyltransferase 1 (DNMT1), DNMT3a, and DNMT3b are upregulated in PRAD tissue compared to normal benign prostatic hyperplastic tissue, and their expression is elevated in cancerous tissue with a higher Gleason score, suggesting a close association between epigenetic alterations and PRAD development and progression (10). Additionally, epigenetic marks are potential biomarkers for PRAD (11) and targets for next-generation drugs.

Copy number variations (CNVs) are the most common genetic alteration in cancers, and CNV burden is associated with the rates of recurrence and death in multiple neoplasms (12). E26 transformation-specific (*ETS*) genes, tumor protein 53 (*TP53*), phosphatase and tensin homolog (*PTEN*), and androgen receptor (*AR*) are the most frequently altered genes in primary prostate cancer, which leads to dysregulation of phosphoinositide 3-kinase (PI3K)/protein kinase B (AKT), RAS/RAF, and cell cycle signaling pathways; moreover, alterations in *AR* and *TP53* have been linked to castration resistance (13, 14) and worse outcomes (15). Thus, CNVs have prognostic value in PRAD as they can reflect disease progression.

The development and progression of cancers involve gene–gene interactions within a gene co-expression network. In this study, we carried out weighted gene co-expression network analysis (WGCNA) (16) to identify genes associated with clinical outcomes in PRAD and can thus serve as biomarkers. We also investigated CNV and methylation status of genes in key module of the network and assessed their prognostic value for PRAD.

## Materials and Methods

### Data Acquisition

The expression data matrix of The Cancer Genome Atlas (TCGA) PRAD database comprising 497 tumor and 52 normal tissue samples along with CNVs, DNA methylation, and clinical information was downloaded from the University of California at Santa Cruz (UCSC) Xena web server (https://xenabrowser.net/).

### Identification of Co-expression Module

Unlike ordinary clustering analysis, clustering criteria of WGCNA have biological significance, so the results obtained by this method have higher credibility. WGCNA clusters genes with similar expression patterns into a module and allows analysis of correlations between module and sample features. In this study, WGCNA was carried out to identify gene module closely related to clinical outcomes in PRAD. To minimize computational burden, the top 10,000 genes with the largest variance were selected. The topological overlap matrix (TOM) was performed to measure the correlation between genes and detection of module, which was able to identify not only the similarity of expression between gene A and gene C, but also the effect of gene A on gene C *via* gene B. A height of 220 in the sample cluster was used to detect outliers, with two outliers as filters. A power β of 8, minimal module size of 30, and branch merge cutoff height of 0.25 were used as the criteria for module construction.

### Copy Number Variation Analysis

The TCGA PRAD CNV profiles were originally measured using whole genome microarray at a TCGA genome characterization center, and GISTIC2 method was then conducted to acquire the estimated values to −2, −1, 0, 1, 2, respectively, representing homozygous deletion, single copy deletion, diploid normal copy, low-level copy number amplification, and high-level copy number amplification (17). The processed data was obtained from https://xenabrowser.net/. In addition, GISTIC2 was conducted to assess the possibility of CNV events in specific chromosomal regions. Genes with changes in frequency >10% were selected for further analysis. We calculated the Spearman correlation coefficient (*r*) between CNVs and gene expression levels, with *r* > 0.4 as the cutoff value, indicating the significant impact on gene expression of CNV.

### Methylation Analysis

The DNA methylation profiles of PRAD from TCGA were available at the University of California, Santa Cruz (UCSC) Xena browser (https://xenabrowser.net/), which were measured experimentally based on the Illumina Infinium HumanMethylation450 platform (Illumina, San Diego, CA, USA). DNA methylation values (β values, between 0 and 1) were recorded for every array probe in each sample by virtue of BeadStudio software (Illumina, San Diego, CA, USA), representing the ratio of the intensity of the methylated bead type to the combined locus intensity. The level of methylation evaluated by β values were derived from the Johns Hopkins University and University of Southern California TCGA genome characterization center.

### Gene Set Enrichment Analysis and Protein–Protein Interaction Network Analysis

Gene Set Enrichment Analysis (GSEA) (18) is a computational method used to determine whether a predefined set of genes can show significant differences between two biological statuses, which were performed by the GSEA software obtained from http://www.broad.mit.edu/gsea to assess the enrichment of identified genes with distinct CNVs and methylation levels in PRAD, with false discovery rate (FDR) <25% and nominal *p* < 0.05 as the cutoff values. Protein–protein interaction (PPI) network analysis of identified genes was completed by an online tool available at https://string-db.org/ to assess possible interactions between their expression products.

### Survival and Statistical Analyses

The Kaplan–Meier survival analysis was performed with Prism 7.0 (GraphPad, La Jolla, CA, USA) and the online tool GEPIA (http://gepia.cancer-pku.cn/index.html) (19). Previous study by Li et al. has established a prognostic model and verified with independent datasets after establishing a prognostic model (20); therefore, we downloaded the independent dataset GSE70769 (21) through the National Center for Biotechnology Information Search database (https://www.ncbi.nlm.nih.gov/) and analyzed the impact of the identified genes on the prognosis of prostate cancer patients. Multivariate analyses were carried out with the cox proportional hazards regression model. All data processing was performed using SPSS v22.0 software (SPSS Inc, Chicago, IL, USA) or R software (x64 3.5.1) (22).

**Figure 1:**
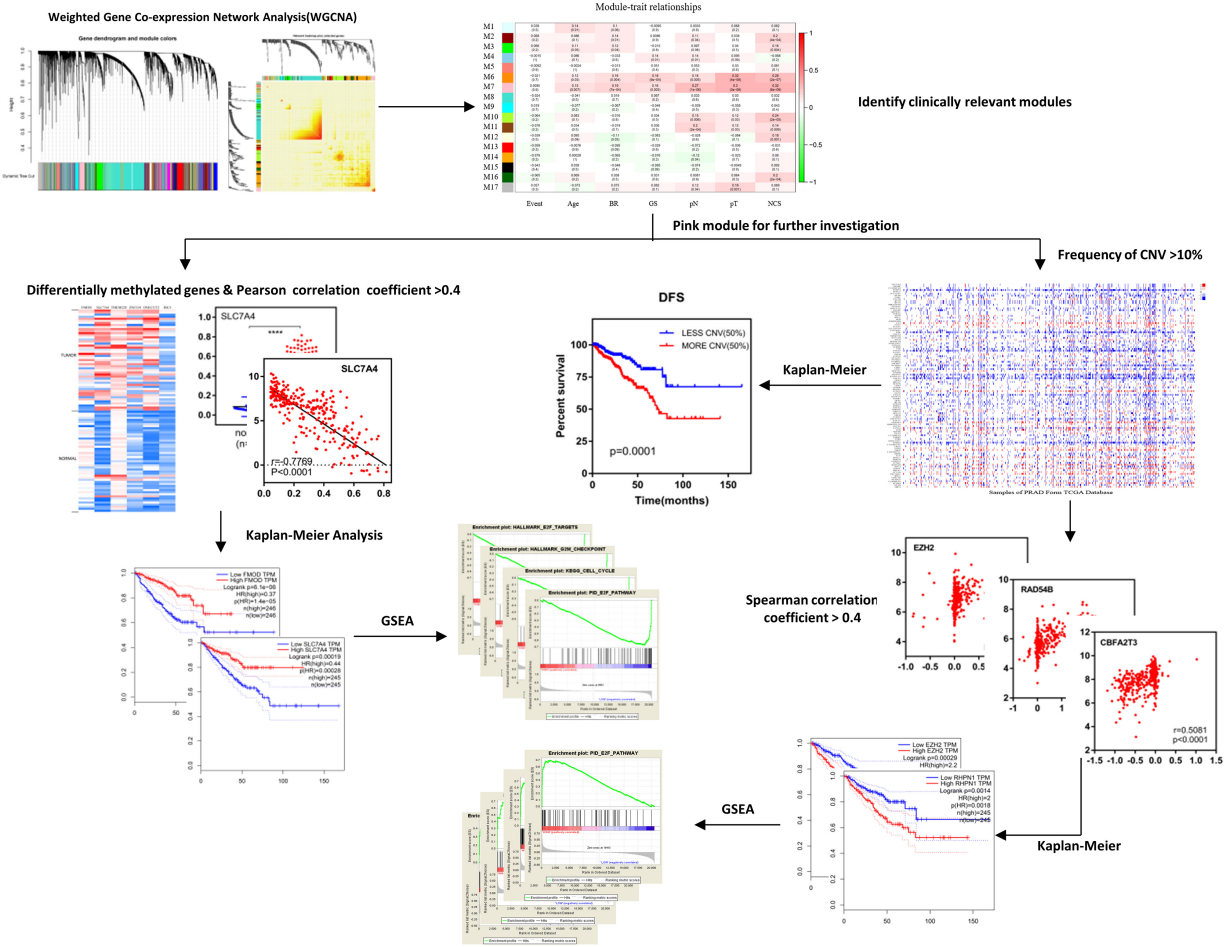
Schematic diagram of the study. After identifying clinically relevant modules with WGCNA, the pink module (M7) was selected for further investigation including differentially methylated genes and frequency of CNVs, afterwards, the Kaplan–Meier survival analysis, and GSEA were used to obtain results and conclusions. WGCNA, weighted gene co-expression network analysis; DFS, disease-free survival; CNV, copy number variation; GSEA, Gene Set Enrichment Analysis.

The research process is illustrated in Figure 1.

## Results

### Identification of Co-expression Module in PRAD

**Figure 2:**
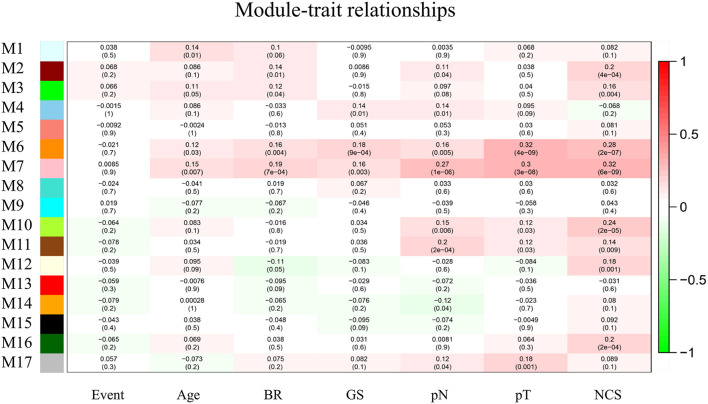
Module–trait relationships. Each column corresponds to one trait, row to one module and every cell contains the correlation coefficient and *p*-value. The gray module represents genes not classified into any module. BR, biochemical recurrence. GS, Gleason score. pN, pathological N stage. pT, pathological T stage.

The top 10,000 genes with the largest variations in expression level relative to normal tissue were selected for WGCNA. We generated a module–trait association network with 7 clinicopathologic traits and 17 modules and calculated the Pearson's correlation coefficients and *p-*values to evaluate the relationship between clinical traits and feature vectors of genes in the module. The module with highest correlation coefficient and module size >30 (pink module, M7, *p* < 0.01) was selected for further analysis (Figure 2).

### Copy Number Variation Analysis

**Figure 3:**
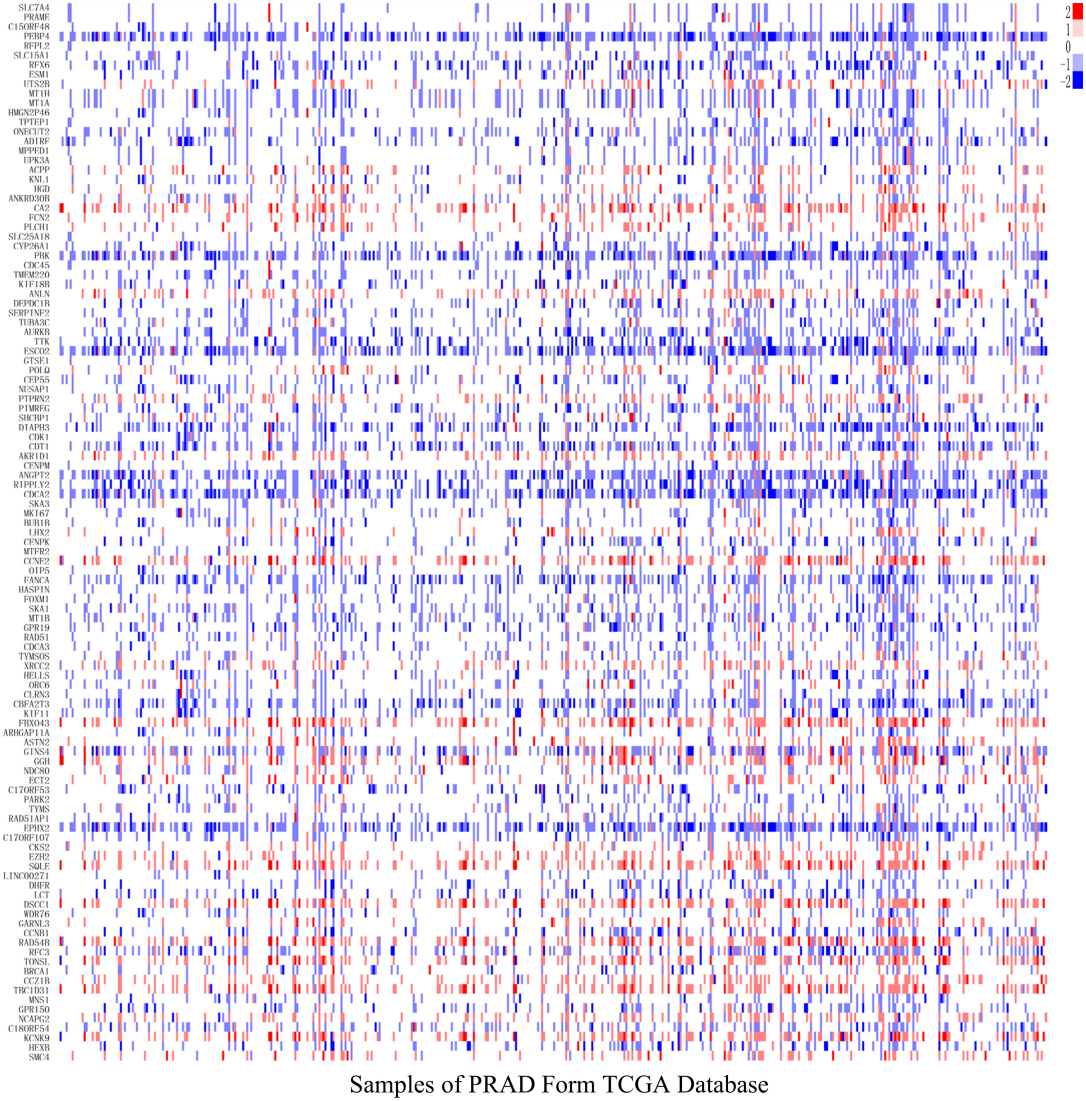
The level of gene copy number in PRAD samples. The estimated values –2, –1, 0, 1, 2, respectively representing homozygous deletion, single copy deletion, diploid normal copy, low-level copy number amplification, and high-level copy number amplification. The horizontal axis represents PRAD tumor samples in TCGA, whereas the vertical axis represents genes from M7 module with CNV > 10%.

**Figure 4:**
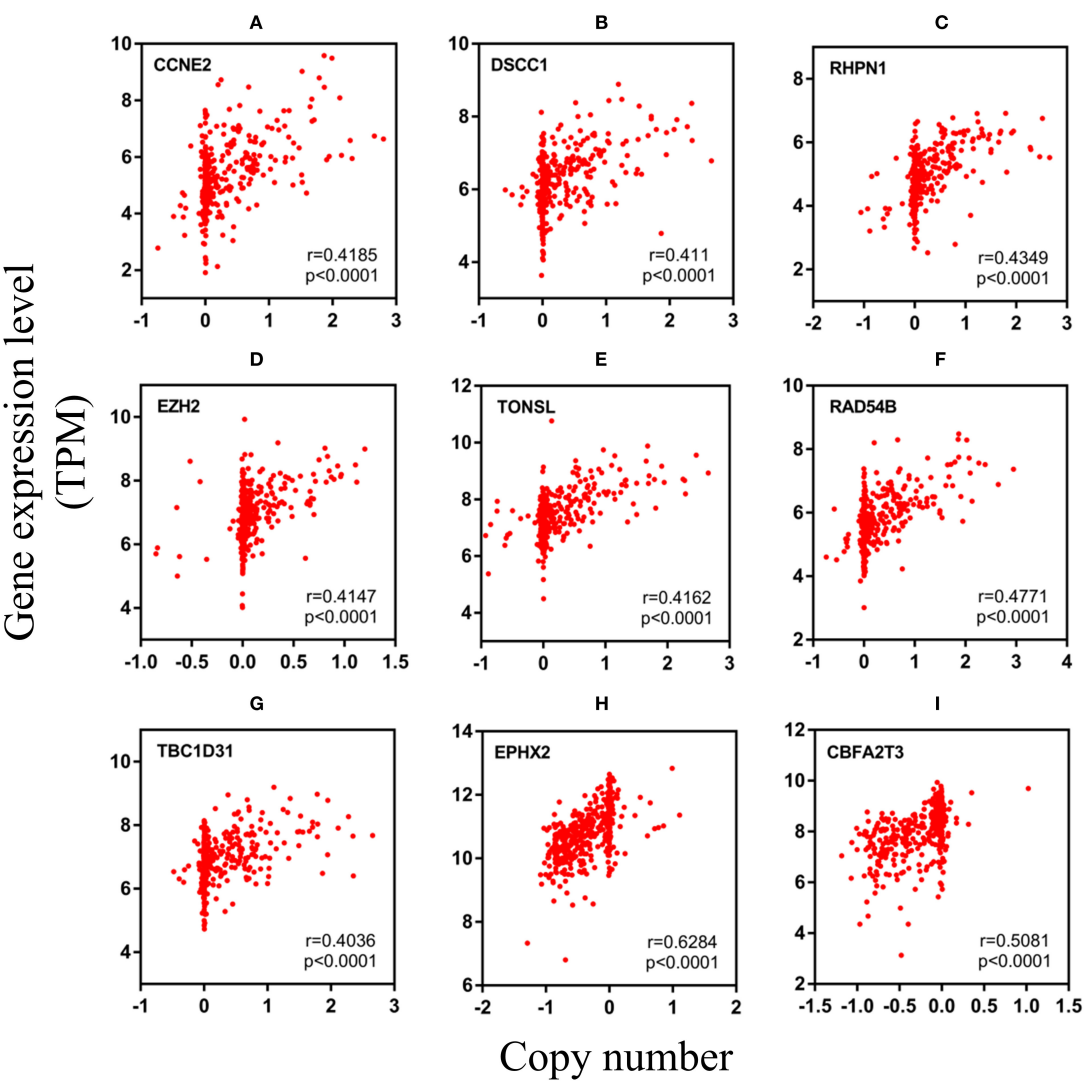
The relevancy of copy number and expression level of nine genes with the Spearman correlation coefficient >0.4. **(A–G)** were mainly manifested as an increase in copy number (amplification) and positively correlated with the level of expression. **(H,I)** were mainly manifested as a loss of copy number (deletion) **(A)** *CCNE2*, **(B)** *DSCC1*, **(C)** *RHPN1*, **(D)** *EZH2*, **(E)** *TONSL*, **(F)** *RAD54B*, **(G)** *TBC1D31*, **(H)** *EPHX2*, and **(I)** *CBFA2T3*. TPM, transcripts per million.

After analyzing CNV profiles of TCGA PRAD data and combining the results with pink module (M7) from the WGCNA, we selected 111 genes with a variation frequency >10% and constructed a heatmap of the CN of genes in the PRAD samples (Figure 3), which allowed us to identify those with abnormal CN. Because our aim was to identify prognostic biomarkers for PRAD, we examined the pathologic stage associated with the CN variants. The 14 genes with the highest CNV and the corresponding clinicopathologic stage are shown in Table 1. Of these, nine genes with a Spearman correlation coefficient >0.4 were selected to evaluate the association between gene CN and expression level. Positive correlations were observed for the cyclin E2 (*CCNE2*), DNA replication and sister chromatid cohesion 1 (*DSCC1*), rhophilin Rho GTPase-binding protein (*RHPN1*), enhancer of zeste homolog 2 (*EZH2*), RAD54B, TBC1 domain family member 31 (*TBC1D31*), and tonsoku-like DNA repair protein (*TONSL*) genes (*p* < 0.0001), indicating the amplifications of CN events probably correlated with higher gene expression level. However, epoxide hydrolase 2 (*EPHX2*) and CBFA2/RUNX1 partner transcriptional co-repressor 3 (*CBFA2T3*) primarily showed deletions of CN events, which leading to the lower level of gene expression (Figure 4). To macro-evaluate the possibility of CNV events in specific chromosomal regions, the deletion and amplification plots based on G scores for CNV were demonstrated in Supplementary Figure 2. The higher G score of a region represents for the greater probability of CNV events in that region.

**Table 1:** Gene copy number variation associated with PRAD clinicopathological staging.

**Gene**		**pT**		***p*-value**		**pN**		***p*-value**
		**pT2**	**pT3+pT4**			**pN0**	**pN1**	
**TMEM220**								
–1	106	24	82		96	71	25	
0	378	162	216	<0.001	319	267	52	0.031
**SQLE**								
0	334	153	181		275	235	40	
1	150	33	117	<0.001	140	103	37	0.003
**RAD54B**								
0	330	153	177		271	231	40	
1	154	33	121	<0.001	144	107	37	0.006
**HEXB**								
–1	89	16	73		82	61	21	
0	395	170	225	<0.001	333	277	56	0.67
**GINS4**								
–1	160	47	113		145	113	32	
0	324	139	185	0.004	270	225	45	0.177
**FBXO43**								
0	325	152	173		266	230	36	
1	159	34	125	<0.001	149	108	41	<0.001
**EZH2**								
0	390	160	230		332	270	62	
1	94	26	68	0.017	83	68	15	0.900
**EPHX2**								
–1	267	73	194		235	184	51	
0	217	113	104	<0.001	180	154	26	0.059
**DSCC1**								
0	335	153	182		276	235	41	
1	149	33	116	<0.001	139	103	36	0.006
**CCNE2**								
0	330	153	177		271	232	39	
1	154	33	121	<0.001	144	106	38	0.003
**CBFA2T3**								
**–**1	189	49	140		169	131	38	
0	295	137	158	<0.001	246	207	39	0.088
**TBC1D31**								
0	334	153	181		275	234	41	
1	150	33	117	<0.001	140	104	36	0.007
**TONSL**								
0	350	156	194		290	246	44	
1	134	30	104	<0.001	125	92	33	0.007
**RHPN1**								
0	350	156	194		290	246	44	
1	134	30	104	<0.001	125	92	33	0.007

*“−1” for deletions and “1” for amplifications*.

### The Kaplan–Meier Survival Analysis

**Figure 5:**
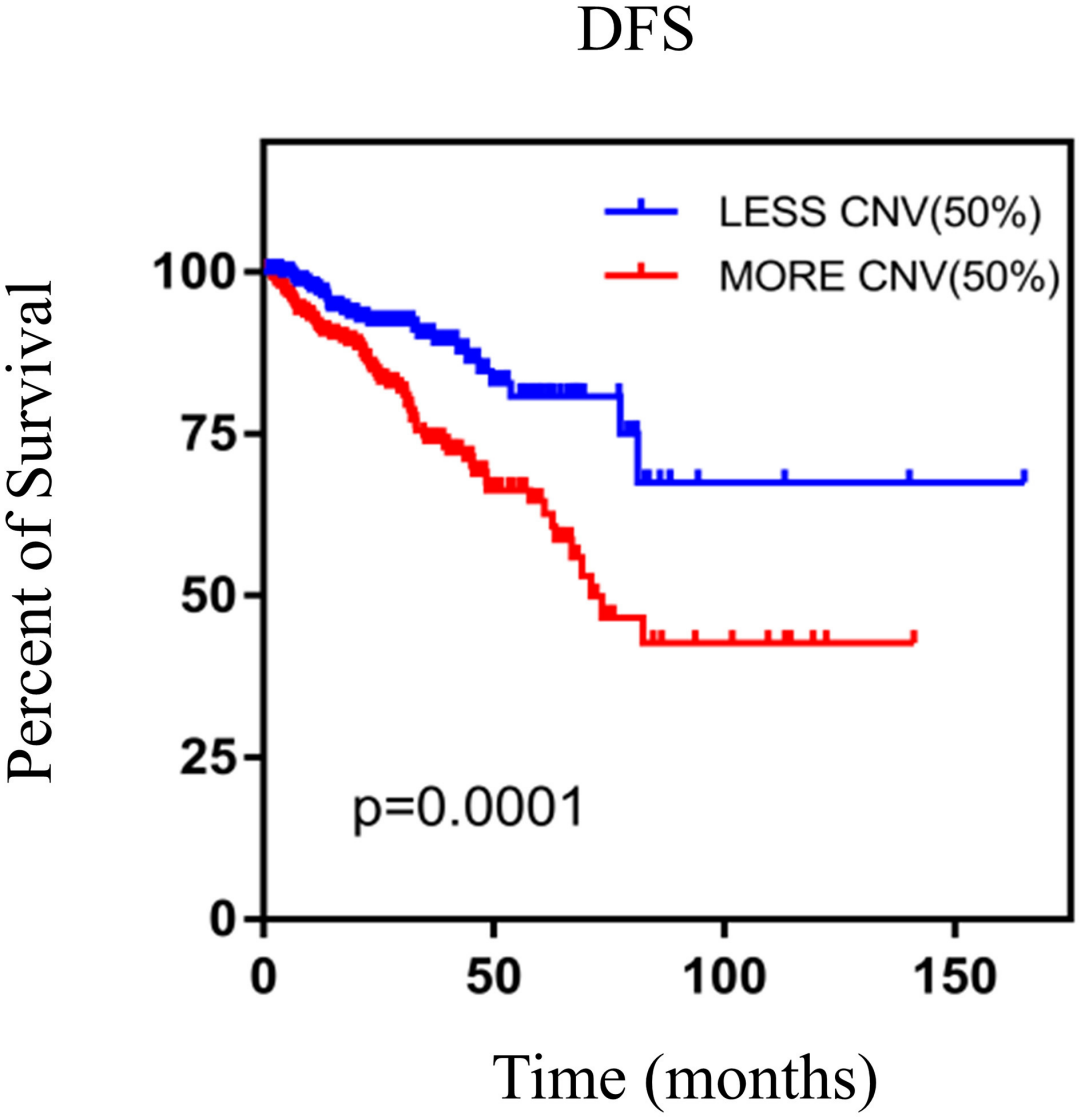
The Kaplan–Meier survival analysis of nine genes. *p* < 0.05 was considered statistically different. DFS, disease-free survival; CNV, copy number variation.

**Figure 6:**
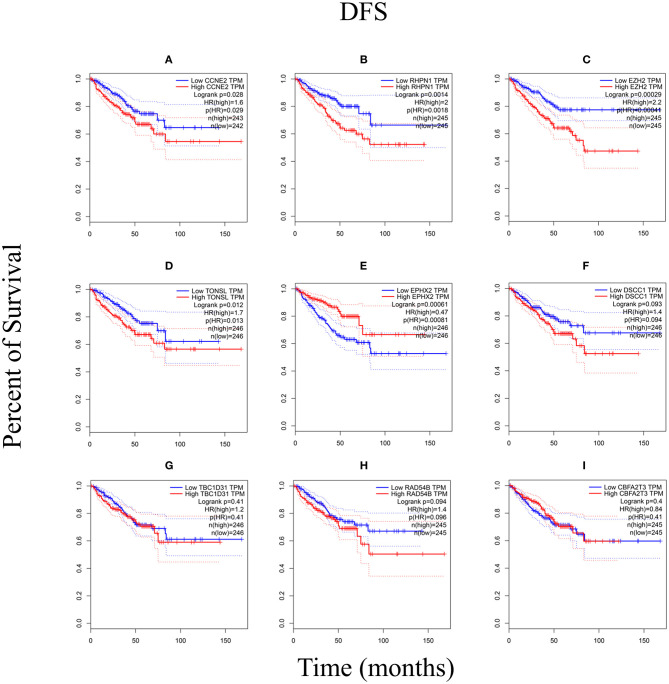
The Kaplan–Meier analysis of nine genes. **(A)** *CCNE2*, **(B)** *RHPN1*, **(C)** *EZH2*, **(D)** *TONSL*, **(E)** *EPHX2*, **(F)** *DSCC1*, **(G)** *TBC1D31*, **(H)** *RAD54B*, and **(I)** *CBFA2T3*. *p* < 0.05 was considered statistically different. DFS, disease-free survival.

We performed the Kaplan–Meier analysis to evaluate the relationship between CNV and disease-free survival (DFS). The survival curve indicated that CNV level was significantly associated with the prognosis of patients with PRAD, with lower CNV predicting longer DFS (*p* = 0.0001; Figure 5). We analyzed the relationship between CNV of the nine above-mentioned genes and patient prognosis and found that lower CNs of *CCNE2* [hazard ratio (HR) = 1.6; *p* < 0.05], *RHPN1* (HR = 2; *p* < 0.05), *EZH2* (HR = 2.2; *p* < 0.001), and *TONSL* (HR = 1.7; *p* < 0.05) were associated with better prognosis, whereas the opposite was true for *EPHX2* (HR = 0.47; *p* < 0.001) (Figure 6). A multivariate analysis of *CBFA2T3* CN suggested that it may be a protective factor in PRAD, whereas the Kaplan–Meier survival analysis suggested it was not statistically significant in the prognosis of patients with PRAD (Figure 6I and Table 2). The validation of identified biomarkers for prognosis value revealed the similar results as our former analysis, indicating the explicit prognostic significance of *CCNE2, SLC7A4, EZH2*, etc. (Supplementary Figures 1B–H).

**Table 2:** Multivariate analysis of CBFA2T3 CNV and patient survival.

**Variable**	**Multivariate analysis**		
	**HR**	**95% CI**	***p*-value**
T stage (≥T3)	2.361	1.175–4.743	0.016
N stage	1.363	0.494–3.726	0.557
M stage	1.254	0.713–2.196	0.457
Gleason score (≥8)	3.172	1.529–6.578	0.002
PSA (≥10)	1.486	0.869–2.432	0.119
CBFA2T3	0.424	0.218–0.824	0.011

*HR estimated from Cox proportional hazard regression model; p < 0.05 was considered statistically significant*.

*DFS, disease-free survival; HR, hazard ratio*.

### Gene Set Enrichment Analysis and PPI Network Analysis

**Figure 7:**
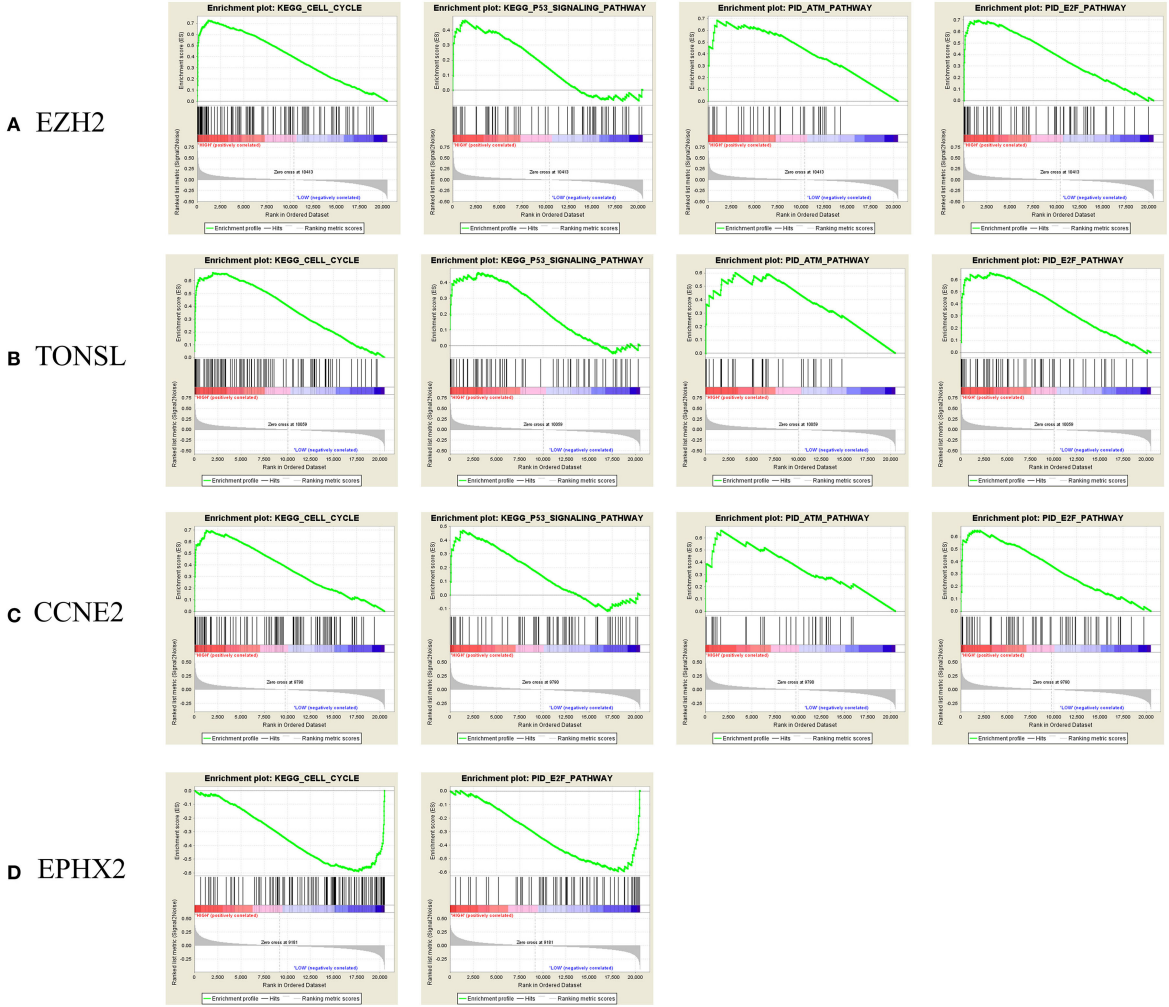
Gene Set Enrichment Analysis (GSEA) curve. **(A)** *E2H2*, **(B)** *TONEL*, **(C)** *CCNE2*, and **(D)** *EPHX-2*. KEGG, Kyoto Encyclopedia of Genes and Genomes. ATM, ataxia telangiectasia-mutated.

To identify enriched gene sets in PRAD samples with high CNV and clarify the mechanisms of CNV in tumorigenesis, we performed GSEA to identify relevant biological pathways in the Kyoto Encyclopedia of Genes and Genomes (KEGG) database and Pathway Interaction Database (PID) using FDR <25% and *p* < 0.05 as the criteria for significance. For *EZH2, TONSL*, and *CCNE2*, the GSEA curves revealed four enriched gene sets including “KEGG–cell cycle,” “KEGG–P53 signaling pathway,” “PID–ataxia–telangiectasia mutated (ATM) pathway,” and “PID–E2F pathway,” which are mainly related to cell cycle regulation, cell apoptosis, and DNA damage repair. Additionally, for *EPHX2*, two functional gene sets were enriched—namely, “Cell cycle pathway” and “PID–E2F pathway” (Figure 7). The PPI network analysis found that there was a co-expression between EZH2 and CCNE2, both of which play important roles in regulating cell cycle (Supplementary Figure 1A).

### Methylation Analysis

**Figure 8:**
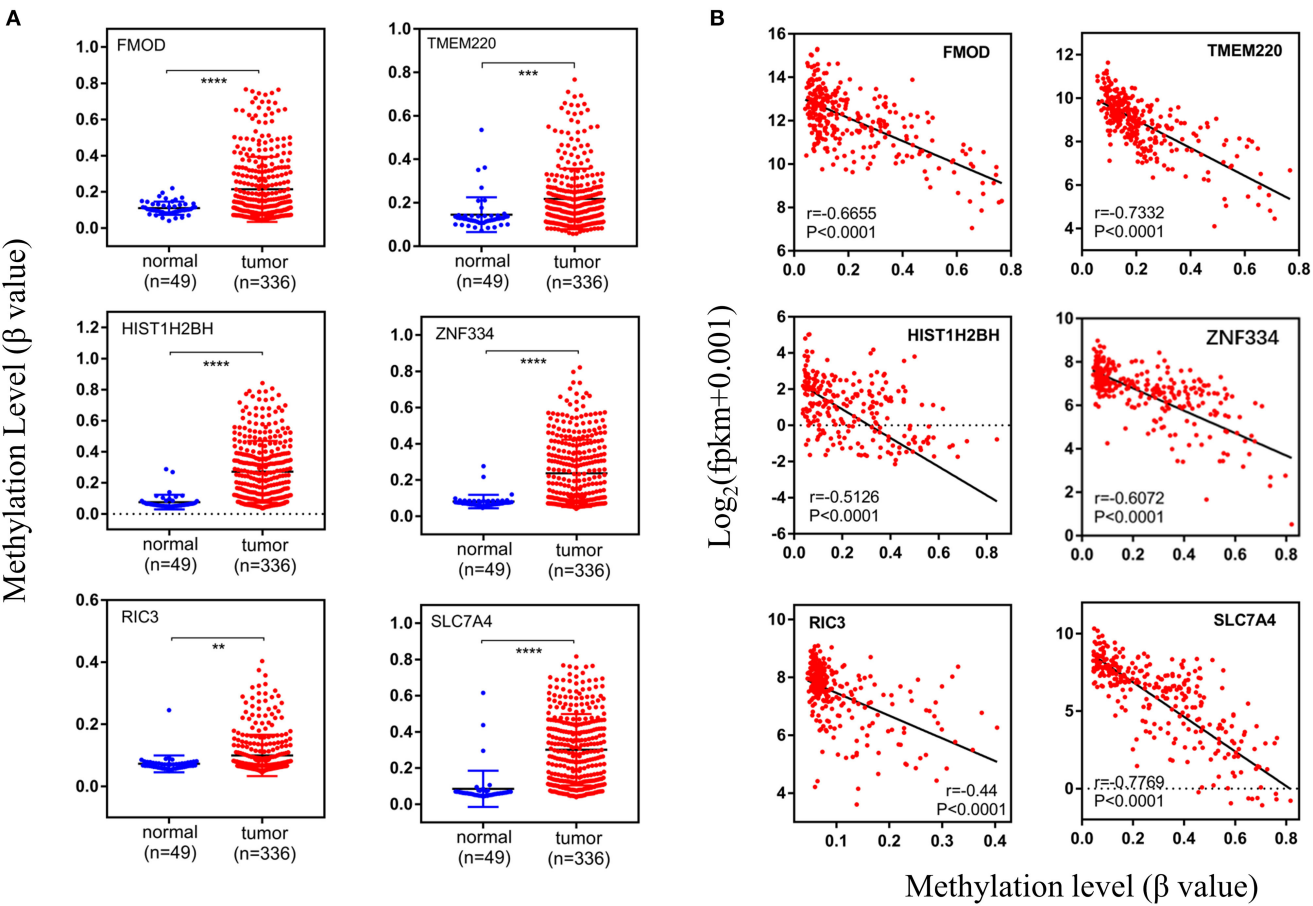
Methylation levels of specific genes. **(A)** The methylation levels in normal and tumor groups, **(B)** the relationship between expression level and methylation level. The vertical axis of **(A)** and the horizontal axis of **(B)** indicate the DNA methylation level (β-value). And *r* represents the Pearson's correlation coefficient, the absolute value closer to 1 means the stronger the correlation. fpkm, fragments per kilobase million.

**Figure 9:**
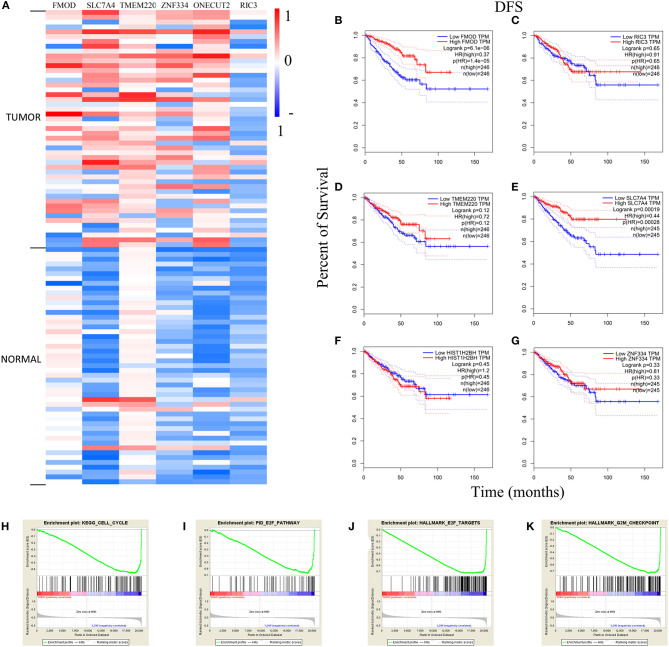
Analysis of genes with high methylation level. **(A)** The differential expression of genes in tumor and normal samples. The Kaplan–Meier survival analysis curves in **(B–G)** demonstrated the relationship between DFS and expression level. **(H–K)** The GSEA curves of *FMOD* and *SLC7A4*. DFS, disease-free survival; GSEA, Gene Set Enrichment Analysis; HR, hazard ratio; TPM, transcripts per million.

After establishing the co-expression module, the DNA methylation level of the genes was examined, and its correlation with gene expression level was evaluated with the Pearson's correlation coefficient. Differentially methylated genes with the Pearson's correlation coefficient >0.4 were identified, including fibromodulin (*FMOD*), transmembrane protein 220 (*TMEM220*), histone H2B type 1-H (*HIST1H2BH*), zinc finger 334 (*ZNF334*), RIC3 acetylcholine receptor chaperone (*RIC3*), and solute carrier family 7 member (*SLC7A4*); these genes were all hypermethylated in tumor samples (*n* = 336) compared to normal tissue (*n* = 49) (Figures 8A, 9A). There was a moderate inverse correlation between gene expression and methylation levels (*r* > 0.4, *p* < 0.001; Figure 8B).

The heatmap of DNA methylation revealed significantly higher levels in tumor tissue compared to normal tissue, especially for *SLC7A4*. The Kaplan–Meier survival curves showed an association between gene expression level and the prognosis of PRAD for *FMOD* [HR (high) = 0.37; *p* < 0.001] and *SLC7A4* [HR (high) = 0.44; *p* < 0.001], with a higher level corresponding to better prognosis (Figures 9B,E), while others were not statistically significant (Figures 9C,D,F,G). The GSEA curves revealed four gene sets that were enriched, including “KEGG–cell cycle,” “PID–E2F pathway,” “Hallmark–E2F target,” and “Hallmark–G2M checkpoint” (Figures 9H–K). These results indicate that *FMOD* and *SLC7A4* are significant genes related to the clinical outcome of PRAD.

## Discussion

The number of new cases of PRAD in the United States has shown an increasing trend in the last 3 years, and PRAD is the second leading cause of death in men despite improvements in diagnostic methods and treatments (2, 23). Although magnetic resonance imaging and some biomarkers are used for the diagnosis of PRAD, the standard approach is tissue biopsy (24), which may only be performed at later stages of the disease when therapeutic options are limited.

Copy number variations occur in 4.8–9.5% of the human genome and play a critical role in tumor recurrence (25); and epigenetic modifications such as DNA methylation are potential biomarkers and targets for treatment in cancer (11). Given the increasing rates of PRAD, there is a need for new diagnostic and prognostic biomarkers with high specificity and sensitivity. In this study, we identified five novel genes with high CNV in PRAD by WGCNA (*CCNE2, RHPN1, EZH2, TONSL*, and *EPHX2*) along with two hypermethylated genes (*FMOD* and *SLC7A4*) that were significantly correlated with the prognosis of patients with PRAD and may thus be clinically useful biomarkers.

Cyclin E2 encodes cyclin E2, a regulatory subunit of cyclin-dependent kinase 2 (CDK2), which controls cell cycle entry from quiescence. Although the gene encoding the other subunit of CDK2, *CCNE1*, has been linked to poor prognosis in hepatocellular carcinoma (HCC), there is little known about the role of *CCNE2* in tumor progression (26). Cyclin E2 was shown to induce the G1-S transition in PC3 prostate cancer cells (27); our results suggest that it may have a similar function in PRAD, given that a lower *CCNE2* CN was associated with longer DFS in patients.

Rhophilin Rho GTPase-binding protein is a Rho GTPase-interacting protein that has not been previously reported in PRAD, but is known to modulate the glomerular filtration barrier and podocyte cytoskeletal architecture (28). The long non-coding RNA *RHPN1* antisense RNA 1 (RHPN1-AS1) was found to promote the progression of several tumors, including uveal melanoma, cervical cancer, and HCC (29–31). Our results provide the first demonstration that overexpression of *RHPN1* is associated with poor prognosis in PRAD.

Enhancer of zeste homolog 2, the catalytic subunit of the DNA methyltransferase polycomb repressive complex 2 (PRC2), is overexpressed in hormone-refractory metastatic PRAD and may be correlated with disease progression and prognosis (32). Consistent with our findings, one study showed that an elevated level of *EZH2* was associated with over proliferation of tumor cells and worse prognosis and may have clinical utility for distinguishing indolent PRAD from aggressive disease with a fatal course (31). On the one hand, the utility of *EZH2* as a biomarker has been demonstrated in patients with intractable PRAD (33). On the other hand, EZH2 inhibitors have been linked to carcinogenesis and treatment resistance in clinical trials (34), though the detailed mechanisms underlying these effects remain to be determined.

Tonsoku-like DNA repair protein promotes homologous recombination during DNA repair in a complex with MMS22-like (MMS22L) (35). However, the role of TONSL in prostate cancer is unknown. We found that a high level of TONSL was associated with enrichment of genes related to the ATM and E2F pathways—which mediate DNA repair and negatively regulate the cell cycle—and decreased survival time in patients with PRAD. Thus, TONSL is a potential biomarker for the progression of PRAD.

Epoxide hydrolase 2 functions in arachidonic acid and androgen signaling (36–38) and has been linked to the biosynthesis and metabolism of cholesterol in the regulation of testosterone levels (39). *EPHX2* silencing induced apoptosis in PRAD cells and enhanced the antiproliferative effect of flutamide (40). In our study, decreased expression of *EPHX2* was associated with the enrichment of genes related to the cell cycle and E2F pathway while patients with an elevated level of *EPHX2* had better prognosis, suggesting that EPHX2 has a protective role in PRAD.

Fibromodulin (encoded by *FMOD*) is thought to be involved in the inhibition of tumorigenesis and apoptosis in hematologic malignancies such as B-cell chronic lymphocytic leukemia and mantle cell lymphoma, like other proteoglycans (40). FMOD was shown to be overexpressed in PRAD cell lines and clinical specimens (41), which is supported by our findings. Our analysis revealed that higher expression of FMOD was associated with a better clinical outcome, highlighting its potential utility as a biomarker for monitoring disease progression.

Solute carrier family 7 member is a cationic amino acid transporter of unknown function; SLC7A4 expressed in the plasma membrane was insufficient to drive amino acid transport (42). Our results showed that *SLC7A4* methylation was higher in tumor specimens than in normal tissue, and that higher *SLC7A4* expression was associated with better clinical outcome. We speculate that SLC7A4 inhibits tumor formation in PRAD through regulation of the cell cycle. Thus, *SLC7A4* likely has clinical value for monitoring PRAD progression and predicting prognosis.

## Conclusion

In this study, we used WGCNA to identify seven genes that are potential prognostic biomarkers for PRAD based on CNV (*CCNE2, RHPN1, EZH2, TONSL*, and *EPHX2*) and DNA hypermethylation (*FMOD* and *SLC7A4*), all of which can serve as indicators of PRAD progression and potential therapeutic targets for the PRAD treatment as well. However, further experiments are needed to elucidate the precise roles and mechanisms of these candidate biomarkers in PRAD and validate their clinical applicability.

## Data Availability Statement

The original contributions presented in the study are included in the article/Supplementary Material, further inquiries can be directed to the corresponding author/s.

## Author Contributions

XY and KC: conception, design of study, and revising the manuscript. YH: drafting the manuscript. JH and LZ: analysis and interpretation of data. YH and LL: acquisition of data. All authors contributed to the article and approved the submitted version.

## Conflict of Interest

The authors declare that the research was conducted in the absence of any commercial or financial relationships that could be construed as a potential conflict of interest.

## Supplementary Material

Supplementary Figure 1 Protein–protein interaction (PPI) and validation of prognosis for identified biomarkers. **(A)** A co-expression between enhancer of zeste homolog 2 (EZH2) and Cyclin E2 (CCNE2), both of which play important roles in regulating cell cycle. **(B–H)** The prognostic value of identified genes in independent dataset GSE70769.

Supplementary Figure 2 The copy number variation (CNV) events in specific chromosomal regions. The higher G score represents for the greater probability of CNV events in that region. The previously identified genes were marked in the figure (*EZH2*: 7q36.1; *CCNE2*: 8q22.1; *RHPN1*: 8q24.3; *TONSL*: 8q24.3; *EPHX2*: 8p21.2-p21.1).

## References

[1] Bray F Ferlay J Soerjomataram I Siegel RL Torre LA Jemal A. Global cancer statistics 2018: GLOBOCAN estimates of incidence and mortality worldwide for 36 cancers in 185 countries. CA Cancer J Clin. (2018) 68:394–424. 10.3322/caac.21492 30207593

[2] Siegel RL Miller KD Jemal A. Cancer statistics, (2020). CA Cancer J Clin. (2020) 70:7–30. 10.3322/caac.21590 31912902

[3] Jones PA Baylin SB. The fundamental role of epigenetic events in cancer. Nat Rev Genet. (2002) 3:415–28. 10.1038/nrg816 12042769

[4] Berger SL Kouzarides T Shiekhattar R Shilatifard A. An operational definition of epigenetics. Genes Dev. (2009) 23:781–3. 10.1101/gad.1787609 19339683 PMC3959995

[5] Jones PA Baylin SB. The epigenomics of cancer. Cell. (2007) 128:683–92. 10.1016/j.cell.2007.01.029 17320506 PMC3894624

[6] Keil KP Vezina CM. DNA methylation as a dynamic regulator of development and disease processes: spotlight on the prostate. Epigenomics. (2015) 7:413–25. 10.2217/epi.15.8 26077429 PMC4500939

[7] Bogdanović O Veenstra GJ. DNA methylation and methyl-CpG binding proteins: developmental requirements and function. Chromosoma. (2009) 118:549–65. 10.1007/s00412-009-0221-9 19506892 PMC2729420

[8] King AD Huang K Rubbi L Liu S Wang CY Wang Y. Reversible regulation of promoter and enhancer histone landscape by DNA methylation in mouse embryonic stem cells. Cell Rep. (2016) 17:289–302. 10.1016/j.celrep.2016.08.083 27681438 PMC5507178

[9] Benetatos L Vartholomatos G. Enhancer DNA methylation in acute myeloid leukemia and myelodysplastic syndromes. Cell Mol Life Sci. (2018) 75:1999–2009. 10.1007/s00018-018-2783-2 29484447 PMC11105366

[10] Gravina GL Ranieri G Muzi P Marampon F Mancini A Di Pasquale B. Increased levels of DNA methyltransferases are associated with the tumorigenic capacity of prostate cancer cells. Oncol Rep. (2013) 29:1189–95. 10.3892/or.2012.2192 23254386

[11] Valdés-Mora F Clark SJ. Prostate cancer epigenetic biomarkers: next-generation technologies. Oncogene. (2015) 34:1609–18. 10.1038/onc.2014.111 24837368

[12] Hieronymus H Murali R Tin A Yadav K Abida W Moller H. Tumor copy number alteration burden is a pan-cancer prognostic factor associated with recurrence and death. Elife. (2018) 7:e37294. 10.7554/eLife.37294.027 30178746 PMC6145837

[13] Robinson D Van Allen EM Wu YM Schultz N Lonigro RJ Mosquera JM. Integrative clinical genomics of advanced prostate cancer. Cell. (2015) 161:1215–28. 10.1016/j.cell.2015.05.001 26000489 PMC4484602

[14] Taylor BS Schultz N Hieronymus H Gopalan A Xiao Y Carver BS. Integrative genomic profiling of human prostate cancer. Cancer Cell. (2010) 18:11–22. 10.1016/j.ccr.2010.05.026 20579941 PMC3198787

[15] Hamid AA Gray KP Shaw G MacConaill LE Evan C Bernard B. Compound genomic alterations of TP53, PTEN, and RB1 tumor suppressors in localized and metastatic prostate cancer. Eur Urol. (2019) 76:89–97. 10.1016/j.eururo.2018.11.045 30553611

[16] Langfelder P Horvath S. WGCNA: an R package for weighted correlation network analysis. BMC Bioinform. (2008) 9:559. 10.1186/1471-2105-9-559 19114008 PMC2631488

[17] Mermel CH Schumacher SE Hill B Meyerson ML Beroukhim R Getz G. GISTIC2.0 facilitates sensitive and confident localization of the targets of focal somatic copy-number alteration in human cancers. Genome Biol. (2011) 12:R41. 10.1186/gb-2011-12-4-r41 21527027 PMC3218867

[18] Subramanian A Kuehn H Gould J Tamayo P Mesirov JP. GSEA-P: a desktop application for gene set enrichment analysis. Bioinformatics. (2007) 23:3251–3. 10.1093/bioinformatics/btm369 17644558

[19] Tang Z Li C Kang B Gao G Li C Zhang Z. GEPIA: a web server for cancer and normal gene expression profiling and interactive analyses. Nucleic Acids Res. (2017) 45:W98–102. 10.1093/nar/gkx247 28407145 PMC5570223

[20] Li R Wang S Cui Y Qu H Chater JM Zhang L. Extended application of genomic selection to screen multiomics data for prognostic signatures of prostate cancer. Brief Bioinform. (2020) bbaa197. 10.1093/bib/bbaa197 32898860

[21] Ross-Adams H Lamb AD Dunning MJ Halim S Lindberg J Massie CM. Integration of copy number and transcriptomics provides risk stratification in prostate cancer: A discovery and validation cohort study. EBioMedicine. (2015) 2:1133-44. 10.1016/j.ebiom.2015.07.017 26501111 PMC4588396

[22] R Core Team. R: A Language and Environment for Statistical Computing. Vienna: R Foundation for Statistical Computing (2013).

[23] Siegel RL Miller KD Jemal A. Cancer statistics, 2019. CA Cancer J Clin. (2019) 69:7–34. 10.3322/caac.21551 30620402

[24] Litwin MS Tan HJ. The diagnosis and treatment of prostate cancer: a review. JAMA. (2017) 317:2532–42. 10.1001/jama.2017.7248 28655021

[25] Zarrei M MacDonald JR Merico D Scherer SW. A copy number variation map of the human genome. Nat Rev Genet. (2015) 16:172–83. 10.1038/nrg3871 25645873

[26] Sonntag R Giebeler N Nevzorova YA Bangen JM Fahrenkamp D Lambertz D. Cyclin E1 and cyclin-dependent kinase 2 are critical for initiation, but not for progression of hepatocellular carcinoma. Proc Natl Acad Sci USA. (2018) 115:9282–7. 10.1073/pnas.1807155115 30150405 PMC6140539

[27] Ye P Shen L Jiang W Ye Y Chen CT Wu X. Zn-driven discovery of a hydrothermal vent fungal metabolite clavatustide C, and an experimental study of the anti-cancer mechanism of clavatustide B. Mar Drugs. (2014) 12:3203–17. 10.3390/md12063203 24879544 PMC4071572

[28] Lal MA Andersson AC Katayama K Xiao Z Nukui M Hultenby K. Rhophilin-1 is a key regulator of the podocyte cytoskeleton and is essential for glomerular filtration. J Am Soc Nephrol. (2015) 26:647–62. 10.1681/ASN.2013111195 25071083 PMC4341472

[29] Lu L Yu X Zhang L Ding X Pan H Wen X. The long non-coding RNA RHPN1-AS1 promotes Uveal melanoma progression. Int J Mol Sci. (2017) 18:226. 10.3390/ijms18010226 28124977 PMC5297855

[30] Duan H Li X Chen Y Wang Y Li Z. LncRNA RHPN1-AS1 promoted cell proliferation, invasion and migration in cervical cancer via the modulation of miR-299-3p/FGF2 axis. Life Sci. (2019) 239:116856. 10.1016/j.lfs.2019.116856 31525429

[31] Fen H Hongmin Z Wei W Chao Y Yang Y Bei L. RHPN1-AS1 drives the progression of hepatocellular carcinoma via regulating miR-596/IGF2BP2 axis. Curr Pharm Des. (2020) 25:4630–40. 10.2174/1381612825666191105104549 31692433

[32] Varambally S Dhanasekaran SM Zhou M Barrette TR Kumar-Sinha C Sanda MG. The polycomb group protein EZH2 is involved in progression of prostate cancer. Nature. (2002) 419:624–9. 10.1038/nature01075 12374981

[33] Xu K Wu ZJ Groner AC He HH Cai C Lis RT. EZH2 oncogenic activity in castration-resistant prostate cancer cells is Polycomb-independent. Science. (2012) 338:1465–9. 10.1126/science.1227604 23239736 PMC3625962

[34] Comet I Riising EM Leblanc B Helin K. Maintaining cell identity: PRC2-mediated regulation of transcription and cancer. Nat Rev Cancer. (2016) 16:803–10. 10.1038/nrc.2016.83 27658528

[35] Duro E Lundin C Ask K Sanchez-Pulido L MacArtney TJ Toth R. Identification of the MMS22L-TONSL complex that promotes homologous recombination. Mol Cell. (2010) 40:632–44. 10.1016/j.molcel.2010.10.023 21055984

[36] Newman JW Morisseau C Harris TR Hammock BD. The soluble epoxide hydrolase encoded by EPXH2 is a bifunctional enzyme with novel lipid phosphate phosphatase activity. Proc Natl Acad Sci USA. (2003) 100:1558–63. 10.1073/pnas.0437724100 12574510 PMC149871

[37] Pinot F Grant DF Spearow JL Parker AG Hammock BD. Differential regulation of soluble epoxide hydrolase by clofibrate and sexual hormones in the liver and kidneys of mice. Biochem Pharmacol. (1995) 50:501–8. 10.1016/0006-2952(95)00167-X 7646556

[38] Tong M Tai HH. Induction of NAD(+)-linked 15-hydroxyprostaglandin dehydrogenase expression by androgens in human prostate cancer cells. Biochem Biophys Res Commun. (2000) 276:77–81. 10.1006/bbrc.2000.3437 11006085

[39] Luria A Morisseau C Tsai HJ Yang J Inceoglu B De Taeye. Alteration in plasma testosterone levels in male mice lacking soluble epoxide hydrolase. Am J Physiol Endocrinol Metab. (2009) 297:E375–83. 10.1152/ajpendo.00131.2009 19458064 PMC2724109

[40] Vainio P Gupta S Ketola K Mirtti T Mpindi JP Kohonen P. Arachidonic acid pathway members PLA2G7, HPGD, EPHX2, and CYP4F8 identified as putative novel therapeutic targets in prostate cancer. Am J Pathol. (2011) 178:525–36. 10.1016/j.ajpath.2010.10.002 21281786 PMC3128506

[41] Reyes N Benedetti I Bettin A Rebollo J Geliebter J. The small leucine rich proteoglycan fibromodulin is overexpressed in human prostate epithelial cancer cell lines in culture and human prostate cancer tissue. Cancer Biomark. (2016) 16:191–202. 10.3233/CBM-150555 26600400 PMC13016539

[42] Wolf S Janzen A Vékony N Martiné U Strand D Closs EI. Expression of solute carrier 7A4 (SLC7A4) in the plasma membrane is not sufficient to mediate amino acid transport activity. Biochem J. (2002) 364:767–75. 10.1042/bj20020084 12049641 PMC1222626

