# Supplementary figures and images for: Integrative Analysis of Methylation and Copy Number Variations of Prostate Adenocarcinoma Based on Weighted Gene Co-expression Network Analysis
Source: Front Oncol. 2021 Apr 1;11:647253. doi: 10.3389/fonc.2021.647253 (PMC8047072; doi:10.3389/fonc.2021.647253)

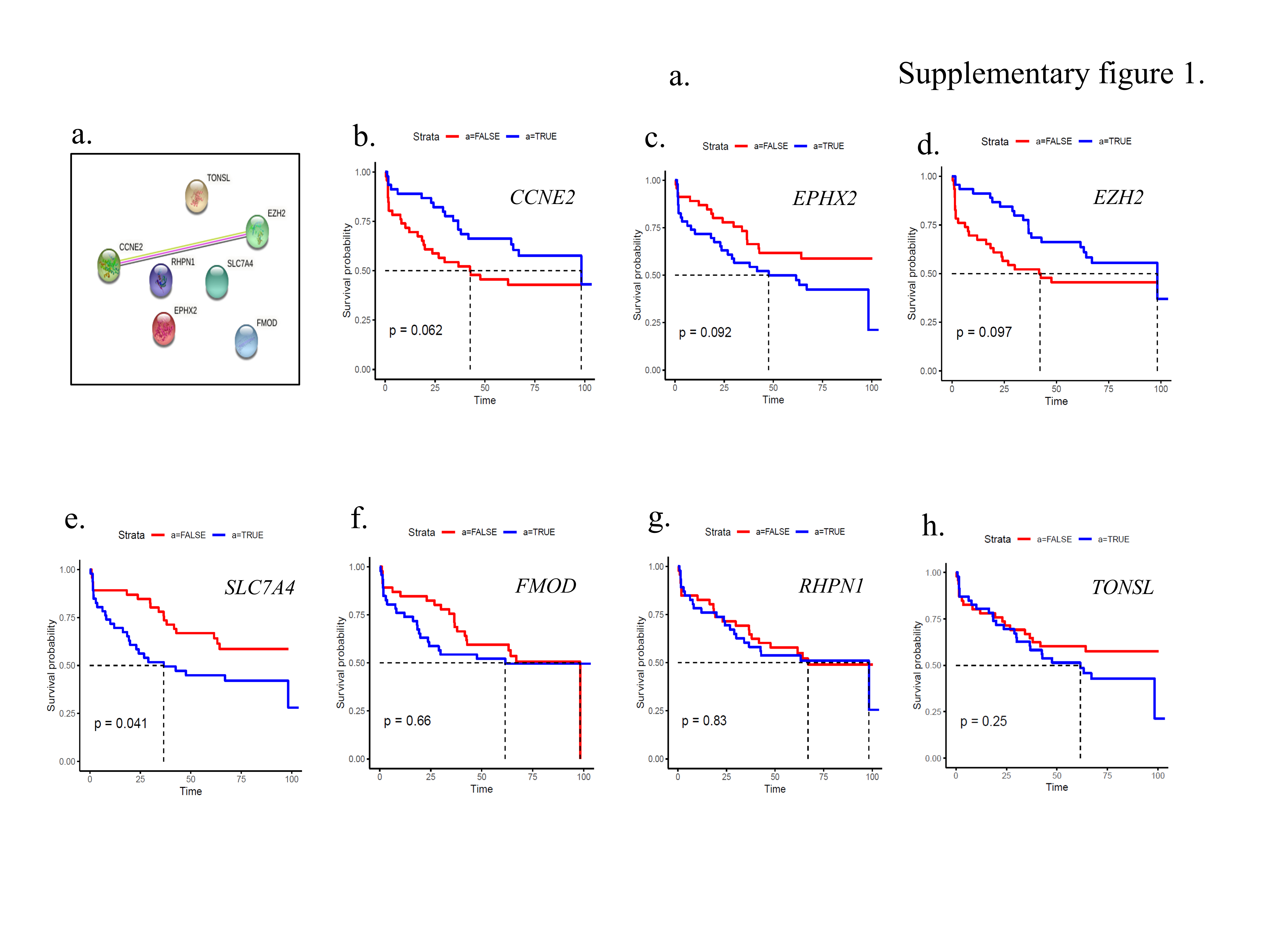

Supplement: Supplementary Figure 1 — Protein–protein interaction (PPI) and validation of prognosis for identified biomarkers. (A) A co-expression between enhancer of zeste homolog 2 (EZH2) and Cyclin E2 (CCNE2), both of which play important roles in regulating cell cycle. (B–H) The prognostic value of identified genes in independent dataset GSE70769. [file Image_1.TIF]

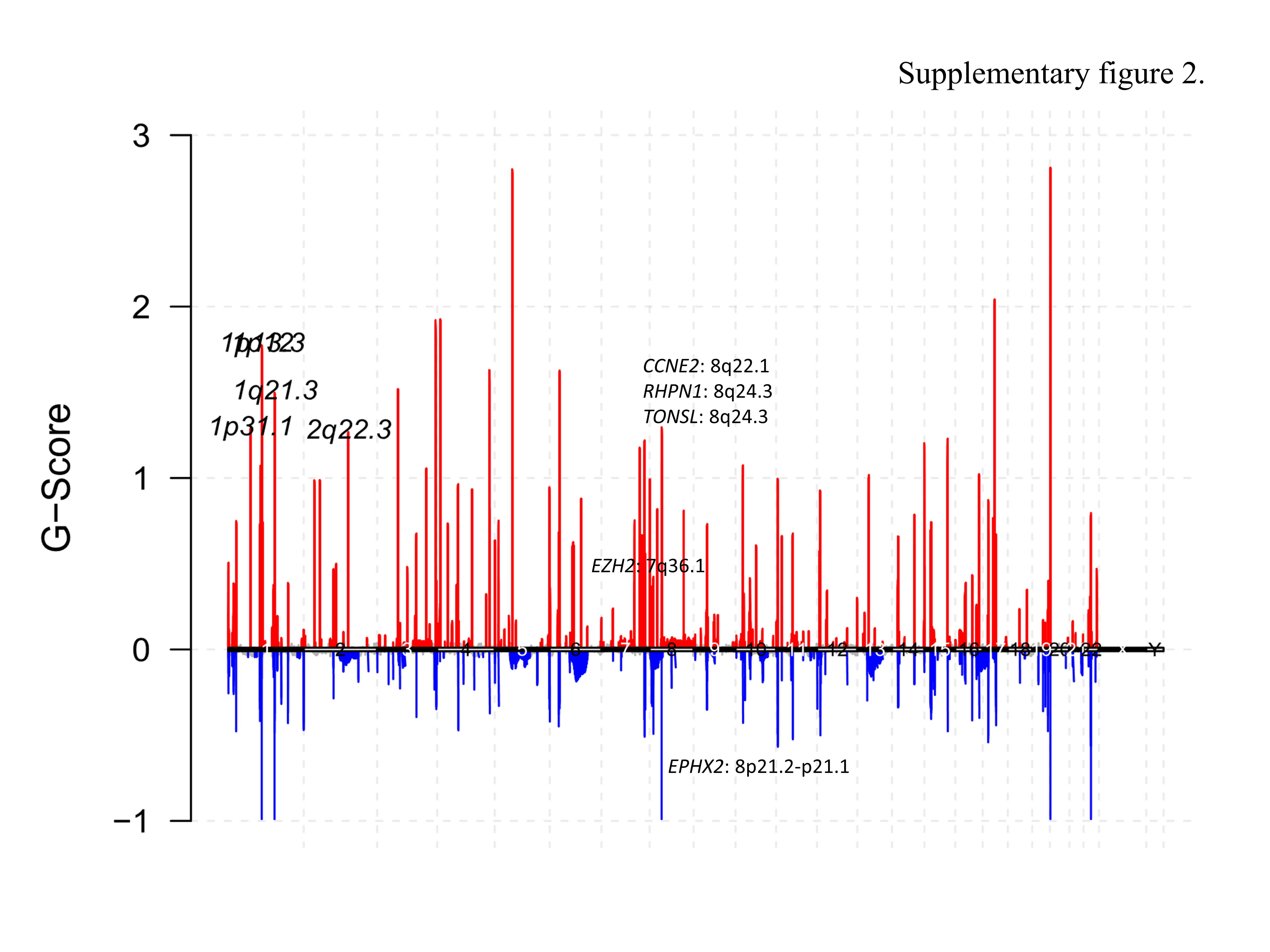

Supplement: Supplementary Figure 2 — The copy number variation (CNV) events in specific chromosomal regions. The higher G score represents for the greater probability of CNV events in that region. The previously identified genes were marked in the figure (EZH2: 7q36.1; CCNE2: 8q22.1; RHPN1: 8q24.3; TONSL: 8q24.3; EPHX2: 8p21.2-p21.1). [file Image_2.TIF]
